# Investigating Psychological Differences Between Nurses and Other Health Care Workers From the Asia-Pacific Region During the Early Phase of COVID-19: Machine Learning Approach

**DOI:** 10.2196/32647

**Published:** 2022-06-01

**Authors:** YanHong Dong, Mei Chun Yeo, Xiang Cong Tham, Rivan Danuaji, Thang H Nguyen, Arvind K Sharma, Komalkumar RN, Meenakshi PV, Mei-Ling Sharon Tai, Aftab Ahmad, Benjamin YQ Tan, Roger C Ho, Matthew Chin Heng Chua, Vijay K Sharma

**Affiliations:** 1 Alice Lee Centre for Nursing Studies Yong Loo Lin School of Medicine National University of Singapore Singapore Singapore; 2 Institute of Systems Science National University of Singapore Singapore Singapore; 3 Dr Moewardi Hospital Surakarta Jawa Tengah Indonesia; 4 Cerebrovascular Disease Department 115 People’s Hospital Ho Chi Minh City Vietnam; 5 Zydus Hospital Ahmedabad India; 6 Yashoda Hospital Secuderabad India; 7 Senthil Multi Specialty Hospital Erode India; 8 University of Malaya Kuala Lumpur Malaysia; 9 Department of Neurology Ng Teng Fong General Hospital Singapore Singapore; 10 Division of Neurology National University Hospital Singapore Singapore; 11 Yong Loo Lin School of Medicine National University of Singapore Singapore Singapore; 12 Department of Psychological Medicine National University Hospital Singapore Singapore

**Keywords:** COVID-19, psychological outcome, machine learning, nurses, health care workers

## Abstract

**Background:**

As the COVID-19 pandemic evolves, challenges in frontline work continue to impose a significant psychological impact on nurses. However, there is a lack of data on how nurses fared compared to other health care workers in the Asia-Pacific region.

**Objective:**

This study aims to investigate (1) the psychological outcome characteristics of nurses in different Asia-Pacific countries and (2) psychological differences between nurses, doctors, and nonmedical health care workers.

**Methods:**

Exploratory data analysis and visualization were conducted on the data collected through surveys. A machine learning modeling approach was adopted to further discern the key psychological characteristics differentiating nurses from other health care workers. Decision tree–based machine learning models (Light Gradient Boosting Machine, GradientBoost, and RandomForest) were built to predict whether a set of psychological distress characteristics (ie, depression, anxiety, stress, intrusion, avoidance, and hyperarousal) belong to a nurse. Shapley Additive Explanation (SHAP) values were extracted to identify the prominent characteristics of each of these models. The common prominent characteristic among these models is akin to the most distinctive psychological characteristic that differentiates nurses from other health care workers.

**Results:**

Nurses had relatively higher percentages of having normal or unchanged psychological distress symptoms relative to other health care workers (n=233-260 [86.0%-95.9%] vs n=187-199 [74.8%-91.7%]). Among those without psychological symptoms, nurses constituted a higher proportion than doctors and nonmedical health care workers (n=194 [40.2%], n=142 [29.5%], and n=146 [30.3%], respectively). Nurses in Vietnam showed the highest level of depression, stress, intrusion, avoidance, and hyperarousal symptoms compared to those in Singapore, Malaysia, and Indonesia. Nurses in Singapore had the highest level of anxiety. In addition, nurses had the lowest level of stress, which is the most distinctive psychological outcome characteristic derived from machine learning models, compared to other health care workers. Data for India were excluded from the analysis due to the differing psychological response pattern observed in nurses in India. A large number of female nurses emigrating from South India could not have psychologically coped well without the support from family members while living alone in other states.

**Conclusions:**

Nurses were least psychologically affected compared to doctors and other health care workers. Different contexts, cultures, and points in the pandemic curve may have contributed to differing patterns of psychological outcomes amongst nurses in various Asia-Pacific countries. It is important that all health care workers practice self-care and render peer support to bolster psychological resilience for effective coping. In addition, this study also demonstrated the potential use of decision tree–based machine learning models and SHAP value plots in identifying contributing factors of sophisticated problems in the health care industry.

## Introduction

In late 2019, well before COVID-19, the World Health Organization (WHO) declared 2020 as the “Year of the Nurse and Midwife” [1]. This was to recognize the vital role of nurses and midwives in health care and their inherent professional challenges, while commemorating the bicentenary of the birth of Florence Nightingale. When the pandemic hit the world in 2020, such recognition was like a fulfilled prophecy. The public are fully aware of the nature, dedication, and challenges of nursing professionals when they risk their lives, together with other health care workers, to fight against COVID-19.

Nurses have been on the frontline, fighting COVID-19, amidst an alarming failure in the global supply of protective gear and new coronavirus tests. Together with unprecedented overwork, global staff shortages have highlighted various vulnerabilities, acknowledged by WHO on the World Health Day.

Nurses play a central role in health care due to the close proximity and amount of time spent with patients. Consequently, they may disproportionately experience ongoing challenges, such as changes in clinical management, shortages of personal protective equipment, work overload, and extended shifts. They may also experience the fear of infection and the emotional toll related to supporting sick and dying patients and their families. As the pandemic evolves, these challenges may impose a significant psychological impact on nurses.

Nursing in 2020 is certainly to be remembered. With approximately 2 years and several COVID-19 waves, the battle against the pandemic seems endless. Health care professionals around the world have been working tirelessly to render support to the health care system embroiled by the pandemic. In particular, the backbone of any health care system—nurses—is enduring and persevering, with no real end of the pandemic in sight.

Several reviews have reported mental health outcomes of health care professionals working during the COVID-19 pandemic. These include stress, anxiety, depression, burnout, and sleep disturbances [2-5]. Particularly within the Asia-Pacific region, both medical and nonmedical health care workers experienced some levels of psychological distress [6]. However, it is unclear whether nurses in this study fared better or worse compared to other health care workers. Several studies have reported that the psychological impacts on nurses were similar to those on other health care workers [7-9]. However, these studies were conducted in China, the epicenter of the initial COVID-19 outbreak. It is unclear whether other Asia-Pacific countries have similar findings.

In view of the lack of evidence on the psychological impacts of COVID-19 on nurses, and to honor nurses and midwives, this study aims to investigate the following: (1) the psychological outcome characteristics of nurses in different Asia-Pacific countries and (2) psychological outcome differences between nurses, doctors, and nonmedical health care workers. This study utilized data from our previous study [10]. The findings may infer focused interventions necessary to address mental health problems amongst health care workers.

## Methods

### Study Population and Study Design

From April 29, 2020, to June 4, 2020, health care workers in major tertiary institutions in India, Indonesia, Malaysia, Singapore, and Vietnam were invited to take part in a survey. Participants included doctors, nurses, and nonmedical health care workers (eg, allied health workers, technicians, administrators). The participating institutions were involved in COVID-19 management during the survey period. The study was approved by the Domain Specific Review Board, National Healthcare Group (2020/00144) as well as the Research Ethics Committee of Zydus Hospitals (2020/220520) and governed by ethical principles of the Declaration of Helsinki.

### Screening Questionnaires

The survey questionnaires included demographic information, medical history, somatic symptoms of participants during the month prior to the study, the Depression Anxiety Stress Scale (DASS-21), and the Impact of Events Scale-Revised (IES-R). These questionnaires were adopted to assess the psychological impact on health care workers due to the COVID-19 outbreak [10].

Depression, anxiety, and stress are the 3 emotional states measured by DASS-21 [11]. DASS-21 is applicable to anyone regardless of their health conditions. Depression, anxiety, and stress scores were derived by adding the scores for questions relevant to each emotional state. DASS-21 comes with thresholds based on the multiplication of emotion scores by 2, which are unique for depression, anxiety, and stress, to categorize levels of severity. DASS-21 was found to have an internal consistency of 0.95 in a psychometric analysis among Chinese hospital workers [12].

Unlike DASS-21, the IES-R measures event-induced distress [13]. It consists of 22 test questions relevant for posttraumatic stress disorder (PTSD) symptoms, namely intrusion, avoidance, and hyperarousal. The score of each symptom was obtained by averaging the scores for relevant test questions. Psychometric analysis amongst Asian populations revealed a Cronbach *α* coefficient for the total scale as .96, while Cronbach *α* for the subscales of intrusion, avoidance, and hyperarousal was .94, .87, and .91, respectively, with a high degree of intercorrelation between the subscales (*r*=0.52-0.87) [14].

### Study Outcomes

We compared the scores of DASS-21 and the IES-R amongst nurses, doctors, and nonmedical health care workers. Similarly, the differences in these scores within a subsample of nurses in different countries were compared. Finally, the most distinctive difference between nurses and other health care professionals was identified.

### Statistical Analysis and Machine Learning Process

**Figure 1:**
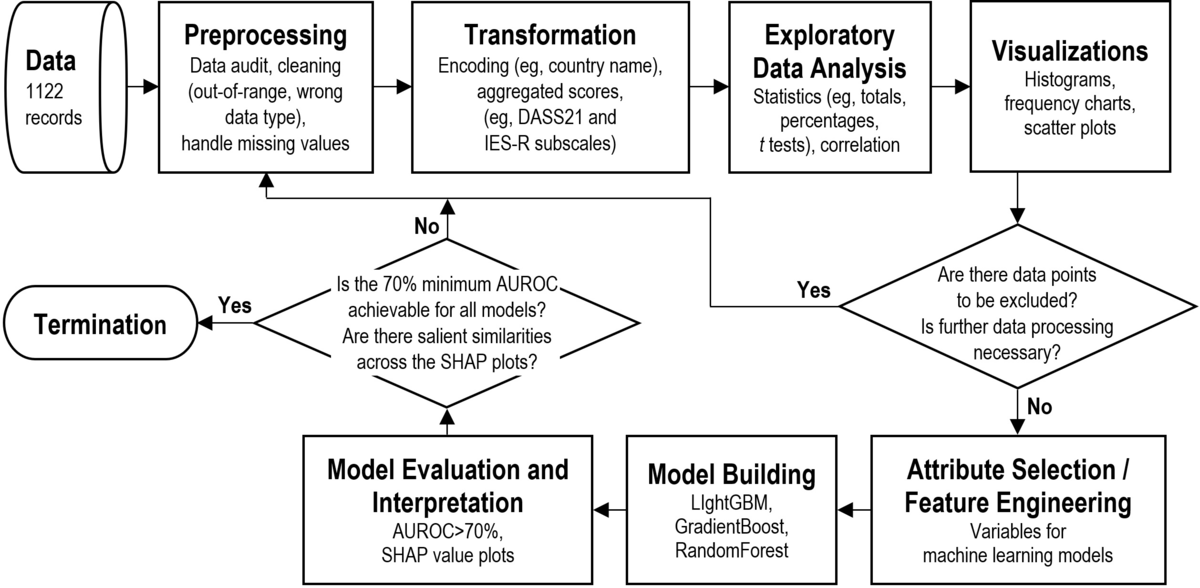
Flow of the analysis process. AUROC: area under the receiver operating characteristic curve; DASS-21: Depression Anxiety Stress Scale; IES-R: Impact of Events Scale-Revised; LightGBM: Light Gradient Boosting Machine; SHAP: Shapley Additive Explanation.

The diagram in Figure 1 depicts the flow of our analysis process.

During transformation (Figure 1), depression, anxiety, stress, intrusion, avoidance, and hyperarousal were expressed as an average value. In the subsequent stages, the average values were used to understand and investigate the psychological outcome characteristics of the various health care professionals and compare them with those of nurses in different countries.

In addition to deciphering useful information from numbers during the exploratory data analysis stage, a visual approach was adopted, as shown in the visualizations stage of the analysis process (Figure 1). Histograms, frequency charts, and scatter plots were used to analyze the spread of hyperarousal mean scores for nurses of each country. As the plots revealed a differing psychological response pattern with obviously higher hyperarousal mean scores for the nurses in India as compared to nurses in other countries, the preprocessing, transformation, exploratory data analysis, and visualizations stages were repeated to prepare another set of statistics excluding the data points for India.

The next step was to identify the critical psychological characteristics that distinguish nurses from other health care workers. Through modeling, the impacts of the psychological variables that made up the model could be discerned. Instead of using traditional mathematical modeling approaches to build the models (eg, probability and statistical models, differential equations, logic models, game theoretic models), machine learning was adopted. Traditional methods are restricted by the underlying theory and assumptions of mathematical models, as well as the need for a mathematician’s expertise to devise the system of mathematical models and inject the model parameter values obtained from calculations. If the underlying mechanisms are misunderstood or incorrect assumptions are made, the derived parameter values will not give rise to adequate goodness of fit. It is a process that relies heavily on human judgement, whereas machine learning methods are not limited by theories and assumptions, and the model parameter values iteratively determined in the course of model training are usually able to produce models that are more accurate than mathematical models. In addition, the verification of mathematical models takes a much longer time as the goal is often to ascertain a worldly theory. Machine learning focuses on finding the association between inputs and outputs, which may not necessarily lead to a conclusion about the causal relationship. The validation of a machine learning model is often not as onerous as the mathematical model and is done right after model training to provide an assurance of the model performance in a timely manner [15-17]. More importantly, the machine learning method suits the objective of this study as the underlying relationships between nurses and their psychological characteristics during COVID-19 were unknown when we began the study. COVID-19 is a novel pandemic that humans have no prior knowledge of. Machine learning methods are commonly used to identify patterns to enhance our understanding of the phenomenon or make predictions about diseases [18].

To identify the most distinctive psychological characteristic of nurses, Shapley Additive Explanation (SHAP) values were extracted from 3 types of decision tree–based models. These steps correspond with the Model Building and Model Evaluation and Interpretation stages of the analysis process (Figure 1). Decision tree–based models were selected as less effort for data preprocessing is required. Normalization and scaling of data values are not necessary for decision tree–based models [19]. Missing values and outliers do not significantly affect the modeling process [19,20]. Even if there are variables that are highly correlated with each other, decision tree–based models are able to handle the multicollinearity [21]. Consideration was also given for their relatively high model accuracy for a small data set upon inherently taking in the interaction effects between variables (interaction terms have to be consciously handled for some model types, eg, regression) [22]. Acceptable models can therefore be quickly built from algorithms for decision tree–based models.

SHAP values are mathematically derived numbers of how much each variable contributes to any machine learning model. The models built were Light Gradient Boosting Machine (LightGBM), GradientBoost, and RandomForest, and they were used for predicting whether a set of psychological distress characteristics belongs to a nurse. A binary target or dependent variable of 1 represented nurses, and 0 represented other health care professionals. The independent variables were depression, anxiety, stress, intrusion, avoidance, and hyperarousal. The data set was balanced through oversampling to achieve equal distribution of data with targets of 1 and 0, before the balanced data set was split into 2 for training and validation purposes in an 80:20 ratio. The parameters for model training were not key in the modeling process. The expectation was not to spend many hours tuning the models for their highest-possible performance but just for the models to be reasonably good for extracting useful information. To ensure that the models were adequately reliable, the target area under the receiver operating characteristic curve (AUROC) of each model, when applied on the validation data set (not requiring a large data set for only testing the AUROC), was set at above 70%. The models were able to converge within a few iterations with the training data set, even with the default algorithm settings. After each model was trained, the model was evaluated using the validation data set. The AUROC of the LightGBM, GradientBoost, and RandomForest models was 73.5%, 78.4%, and 74.0%, respectively. Subsequently, the rank of influence of each variable was concluded visually from the SHAP values for the training data set. The interpretations of the SHAP value plots are discussed in the Results section. The common-most influential variable across all the models, akin to the distinctive psychological outcome characteristic of nurses, as compared to other health care professionals, was established. This technique was used to discover the unique qualities of the K-Pop group Bangtan Boys (BTS) [23]. All analyses were performed in Python 3.7.3 with Anaconda Jupyter Notebook and SHAP version 0.37.0.

It is always necessary to review the steps taken in data preprocessing and transformation if the accuracy of models is not acceptable or no conclusion can be drawn from the SHAP value plots. The decision symbol connected to the Model Evaluation and Interpretation stage in Figure 1 depicts the feedback loop to initial stages. We did not observe consistencies in the SHAP value plots in our first attempt. We reperformed data preprocessing to remove data for India due to the differing psychological response pattern of the nurses in India, before we were able to observe an obvious similarity across all SHAP value plots.

## Results

### Participant Characteristics

**Figure 2:**
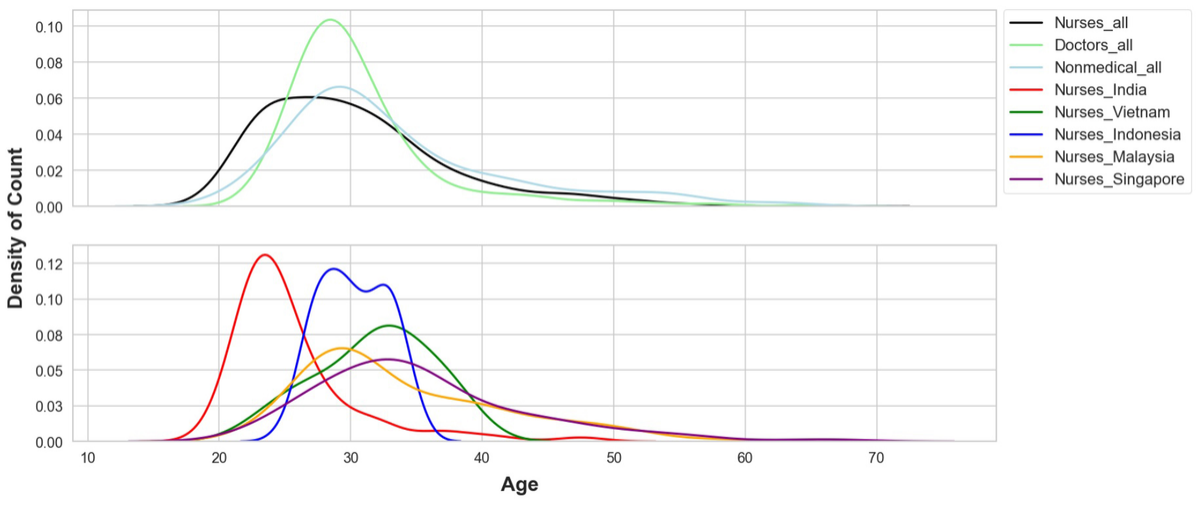
Age distribution (overall sample vs nurses subsample).

A total of 1122 participants, including doctors, nurses, and nonmedical health care workers, were recruited from India, Indonesia, Malaysia, Singapore, and Vietnam. Nurses comprised 39.0% (n=438) of the study population, followed by nonmedical health care workers (n=389, 34.7%) and doctors (n=295, 26.3%). The median (IQR) age was 30 (27-34) years, with most participants being female (n=732, 65.2%) and married (n=606, 54%). The majority of the participants were Indian (n=436, 38.9%); see Table 1. Amongst the nurses (n=438, 39.0%), there was a significantly higher proportion of female nurses as compared to male nurses (362 [82.6%] vs 76 [17.4%]); see Table 2. In addition, nurses were relatively younger than other health care professions due to the relatively lower median age (24 years) amongst nurses in India (Figure 2).

**Table 1:** Baseline characteristics of study participants (N=1122).

Characteristic		Value
Age (years), median (IQR)		30 (27-34)
**Type of profession, n (%)**		
	Nurses	438 (39.0)
	Doctors	295 (26.3)
	Nonmedical	389 (34.7)
**Country, n (%)**		
	India	384 (34.2)
	Malaysia	175 (15.6)
	Singapore	254 (22.6)
	Indonesia	249 (22.2)
	Vietnam	60 (5.4)
**Sex, n (%)**		
	Female	732 (65.2)
	Male	390 (34.8)
**Ethnicity, n (%)**		
	Indian	434 (38.7)
	Malay	211 (18.8)
	Chinese	154 (13.7)
	Others	323 (28.8)
**Marital status, n (%)**		
	Married	606 (54.0)
	Single	493 (44.0)
	Divorced, separated, or widowed	23 (2.0)

**Table 2:** Baseline characteristics of nurses (N=438).

Characteristic		Value
Age (years), median (IQR)		29 (25.0-33.5)
**Country, n (%)**		
	India	167 (38.1)
	Malaysia	94 (21.5)
	Singapore	93 (21.2)
	Indonesia	64 (14.6)
	Vietnam	20 (4.6)
**Sex, n (%)**		
	Female	362 (82.6)
	Male	76 (17.4)
**Ethnicity, n (%)**		
	Indian	179 (40.9)
	Malay	84 (19.2)
	Chinese	43 (9.8)
	Others	132 (30.1)
**Marital status, n (%)**		
	Married	218 (49.8)
	Single	211 (48.2)
	Divorced, separated, or widowed	9 (2.0)

### Psychological Characteristics of Nurses in Different Countries

Considering psychological distress characteristics of nurses only, except for hyperarousal and anxiety, all other psychological distress scores were highest among nurses in Vietnam. Nurses in India and Singapore exhibited highest levels of hyperarousal and anxiety, respectively (Table 3).

**Figure 3:**
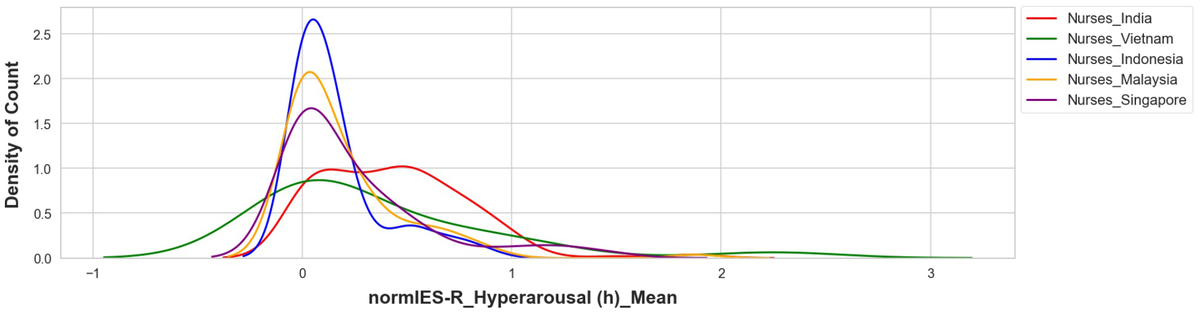
Hyperarousal of nurses by country (overlapping distribution plots). IES-R: Impact of Events Scale-Revised.

**Figure 4:**
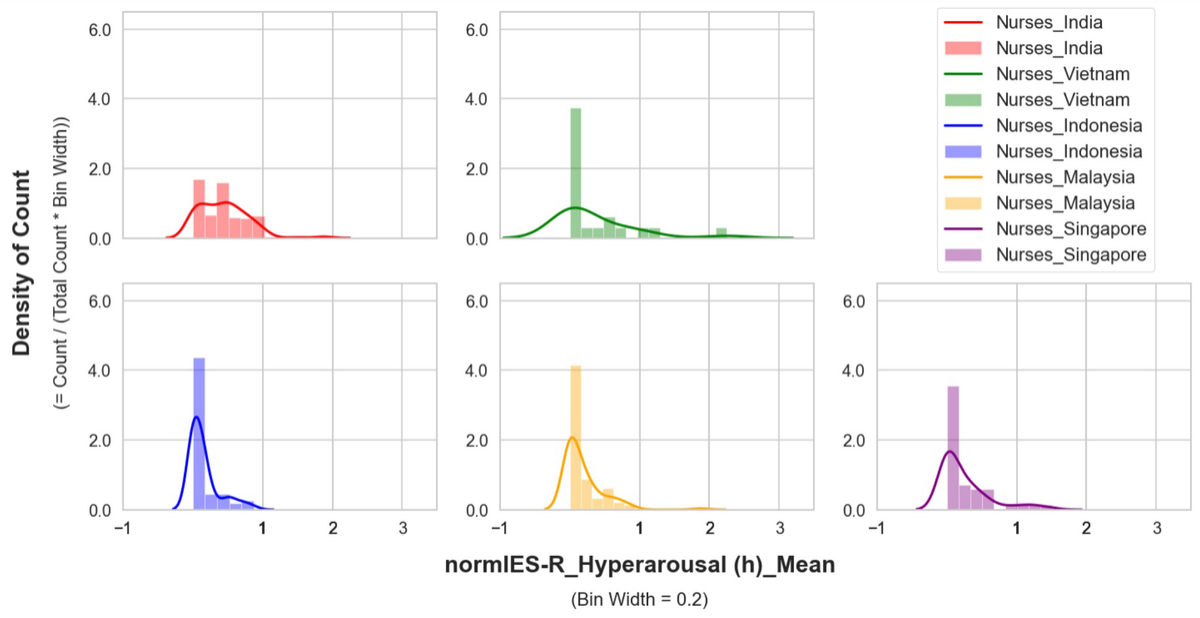
Hyperarousal of nurses by country (plots with the same y axis limit). IES-R: Impact of Events Scale-Revised.

**Figure 5:**
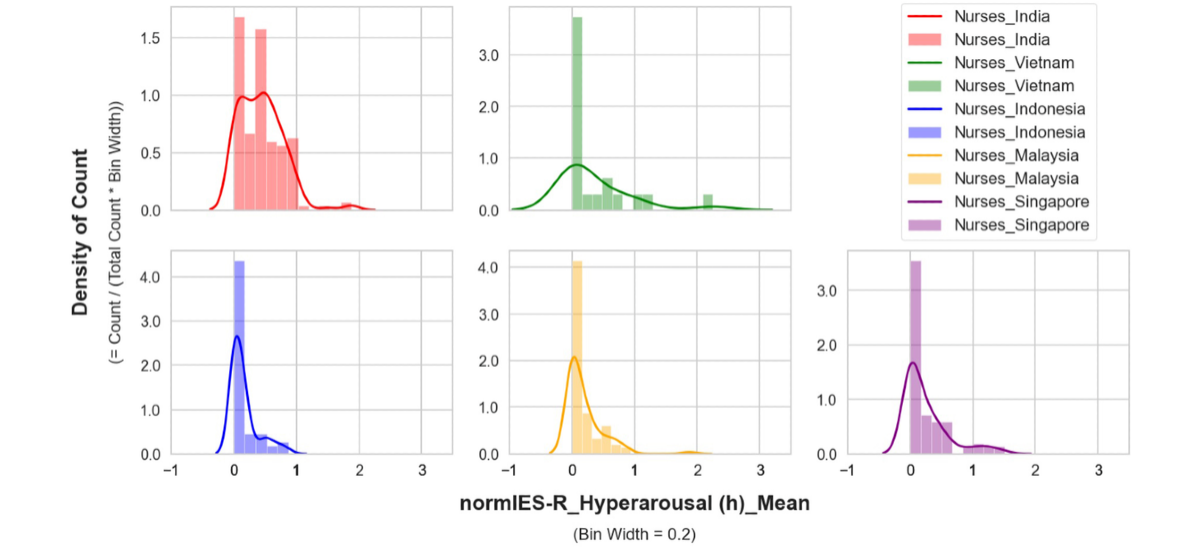
Hyperarousal of nurses by country (plots with different y axis limits). IES-R: Impact of Events Scale-Revised.

**Figure 6:**
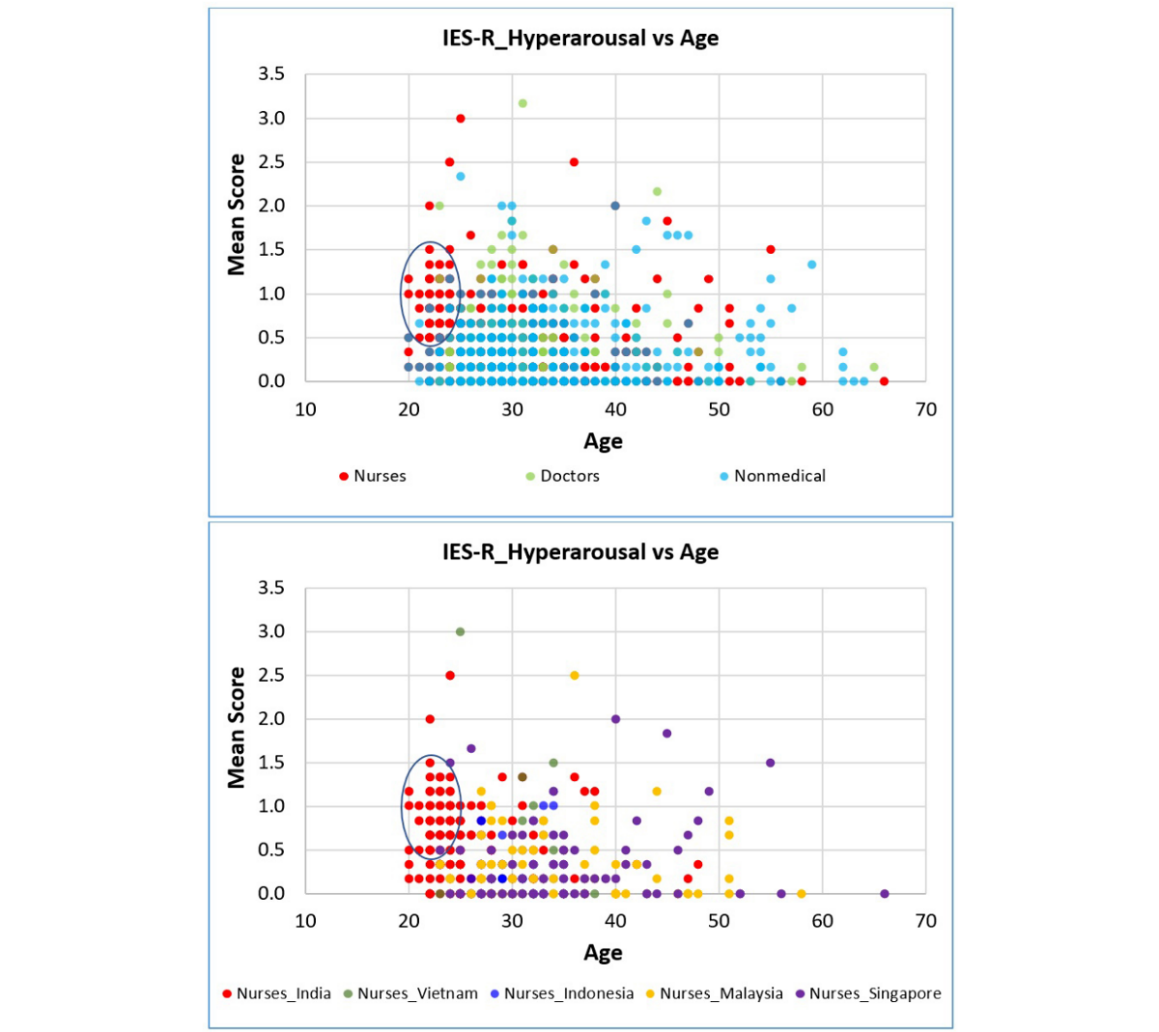
Hyperarousal versus age, by profession and by nurses of each country (dots inside circles represent younger nurses in India). IES-R: Impact of Events Scale-Revised.

The mean distribution of hyperarousal scores in the nurses showed that their overall higher hyperarousal scores were mainly due to the scores of nurses in India (Figures 3-5). A higher count density for hyperarousal mean values and 2 peaks of density (at mean values between 0.0 and 0.2 and between 0.6 and 1.0) in the distribution were observed in the nurses in India (more clearly shown in Figure 5). This was attributed to younger nurses in India (aged 20-24 years) having higher hyperarousal scores (Figure 6) relative to their same-age peers (ie, other health care workers or nurses) from other countries. Based on this observation, it became apparent that the nurses in India had a psychological response pattern that was different from the nurses in the other 4 countries.

Upon excluding the data for India, nurses in Vietnam had the highest scores for all psychological distress characteristics, except for anxiety. Nurses in Singapore showed the highest anxiety score (Table 3).

**Table 3:** Mean scores^a^ of psychological distress characteristics for nurses by country (all 5 countries, including India).

Country	Depression	Anxiety	Stress	Intrusion^b^	Avoidance^b^	Hyperarousal^b^
India	0.1566	0.1575	0.2439	0.2470	*0.2869*	*0.4386*
Indonesia	0.1273	0.1720	0.2278	*0.3647*	0.2798	0.1543
Malaysia	0.1596	0.1657	0.2234	0.2294	0.2424	0.1915
Singapore	*0.2167*	*0.2320*	*0.2827*	*0.2984*	0.2772	0.2460
Vietnam	*0.3286*	*0.1930*	*0.3787*	*0.3984*	*0.4875*	*0.3625*
Overall mean	0.1736	0.1788	0.2515	0.2783	0.2834	0.2997

^a^The average of mean scores or normalized mean scores.

^b^Normalized values by multiplying by 3 and dividing by 4 were adopted for IES-R (Impact of Events Scale-Revised) subscales (ie, intrusion, avoidance, and hyperarousal). This was to make IES-R scores (0-4) to be in the same scale as DASS-21 (Depression Anxiety Stress Scale) (0-3).

^c^Numbers in italics are the mean scores of nurses in different countries that are higher than their respective overall mean scores (last row of the table).

### Differences Between Nurses, Doctors, and Nonmedical Health Care Workers

As discussed in the previous section that the nurses in India exhibited a vastly different psychological response pattern, and the data for India were excluded from the subsequent analysis and building of machine learning models (analysis including the data points for India is provided in Tables S1 and S2 of Multimedia Appendix 1, corresponding to Tables 4 and 5 of this main paper, respectively). Upon excluding the nurses in India, the overall mean hyperarousal score of nurses reduced to 0.2140 (normalized), which was below the overall sample mean of 0.2463 (normalized); see Table 4. This, in turn, placed the nurses’ hyperarousal score to be the lowest. Similarly, the scores for depression, anxiety, stress, intrusion, and avoidance amongst nurses were below the respective overall sample mean (Table 4). The most severe levels of psychological distress were observed in nonmedical health care workers, with the highest scores for all psychological distress characteristics except for depression. Doctors were affected in terms of depression (0.3200) and stress (0.4424) during the early COVID-19 phase (Table 4).

Regarding the clinical severity of psychological outcomes, most participants displayed normal or unchanged psychological distress characteristics (n=609-666, 82.5%-91.2%); see Table 5. Nurses, however, had higher percentages of normal or unchanged characteristics compared to other health care workers: in nurses, avoidance was the lowest (n=233, 86.0%) and stress was the highest (n=260, 95.9%), while among other nonnurse workers, avoidance was the lowest (n=187, 74.8%) in nonmedical health care workers and hyperarousal was the highest (n=199, 91.7%) in doctors (see Table 5). Thus, nurses were least psychologically affected by COVID-19.

There were 482 participants who were normal or had no symptoms at all for all the 6 psychological distress characteristics. Among them, there were 194 (40.2%) nurses, followed by 146 (30.3%) nonmedical health care workers and 142 (29.5%) doctors.

**Table 4:** Mean scores^a^ of psychological distress characteristics by profession and *t* test results (4 countries, not including India).

Profession		Depression	Anxiety	Stress	Intrusion^b^	Avoidance^b^	Hyperarousal^b^
**Scores by profession**							
	Overall	0.2546	0.2577	0.3775	0.3360	0.3363	0.2463
	Nurses	0.1840	0.1920	0.2562	0.2975	0.2813	0.2140
	Doctors	*0.3200^c^*	*0.2792*	*0.4424*	0.3085	0.3020	0.2310
	Nonmedical	*0.2743*	*0.3104*	*0.4526*	*0.4016*	*0.4256*	*0.2945*
**Two-tailed *P* values of a two-sample *t* test for comparing mean values (Cronbach *α*=.05)**							
	Nurses vs doctors	<.001 (diff^d^)	.005 (diff)	<.001 (diff)	.75 (no diff^d^)	.57 (no diff)	.55 (no diff)
	Nurses vs nonmedical	.009 (diff)	<.001 (diff)	<.001 (diff)	.004 (diff)	<.001 (diff)	.008 (diff)

^a^The average of mean scores or normalized mean scores.

^b^Normalized values by multiplying by 3 and dividing by 4 were adopted for IES-R (Impact of Events Scale-Revised) subscales (ie, intrusion, avoidance, and hyperarousal). This was to make IES-R scores (0-4) to be in the same scale as DASS-21 (Depression Anxiety Stress Scale) (0-3).

^c^Numbers in italics are the mean scores by profession that are higher than their respective overall mean scores (first row of the table).

^d^If the *P* value of the 2-sample *t* test was <.05, it represented that there was a difference in the mean scores or normalized mean scores (denoted by “diff”). Otherwise, there was no difference (denoted by “no diff”).

**Table 5:** Psychological distress severity (4 countries, not including India).^a^

Severity category		Depression, n (%)	Anxiety, n (%)	Stress, n (%)	Intrusion, n (%)	Avoidance, n (%)	Hyperarousal, n (%)
**All (nurses, doctors, and nonmedical health care workers; N=738)**							
	Normal/not at all	638 (86.4)	614 (83.2)	673 (91.2)	627 (85.0)	609 (82.5)	666 (90.2)
	Mild/a little bit, and above	100 (13.5)	124 (16.8)	65 (8.8)	111 (15.0)	129 (17.5)	72 (9.8)
**Nurses (N=271)**							
	Normal/not at all	245 (90.4)	242 (89.3)	260 (95.9)	234 (86.4)	233 (86.0)	249 (91.9)
	Mild/a little bit, and above	26 (9.6)	29 (10.7)	11 (4.1)	37 (13.6)	38 (14.0)	22 (8.1)
**Doctors (N=217)**							
	Normal/not at all	178 (82.0)	173 (79.7)	193 (88.9)	191 (88.0)	189 (87.1)	199 (91.7)
	Mild/a little bit, and above	39 (18.0)	44 (20.3)	24 (11.1)	26 (12.0)	28 (12.9)	18 (8.3)
**Nonmedical health care workers (N=250)**							
	Normal/not at all	215 (86.0)	199 (79.6)	220 (88.0)	202 (80.8)	187 (74.8)	218 (87.2)
	Mild/a little bit, and above	35 (14.0)	51 (20.4)	30 (12.0)	48 (19.2)	63 (25.2)	32 (12.8)

^a^DASS-21 (Depression Anxiety Stress Scale) severity categories are based on 2 times of the sum of subscale scores: depression mild and above, ≥10; anxiety mild and above, ≥8; stress mild and above, ≥15. IES-R (Impact of Events Scale-Revised) severity categories are based on the mean of subscale scores: intrusion, avoidance, and hyperarousal, a little bit and above, ≥1.

### Distinctive Psychological Distress Characteristics of Nurses

Due to the differing psychological response pattern of nurses in India, all data for India were excluded to build the 3 decision tree–based machine learning models (LightGBM, GradientBoost, and RandomForest) for predicting whether a health care worker is a nurse, based on the psychological scores. The SHAP values of these models were extracted to identify the distinctive psychological distress characteristics of nurses.

**Figure 7:**
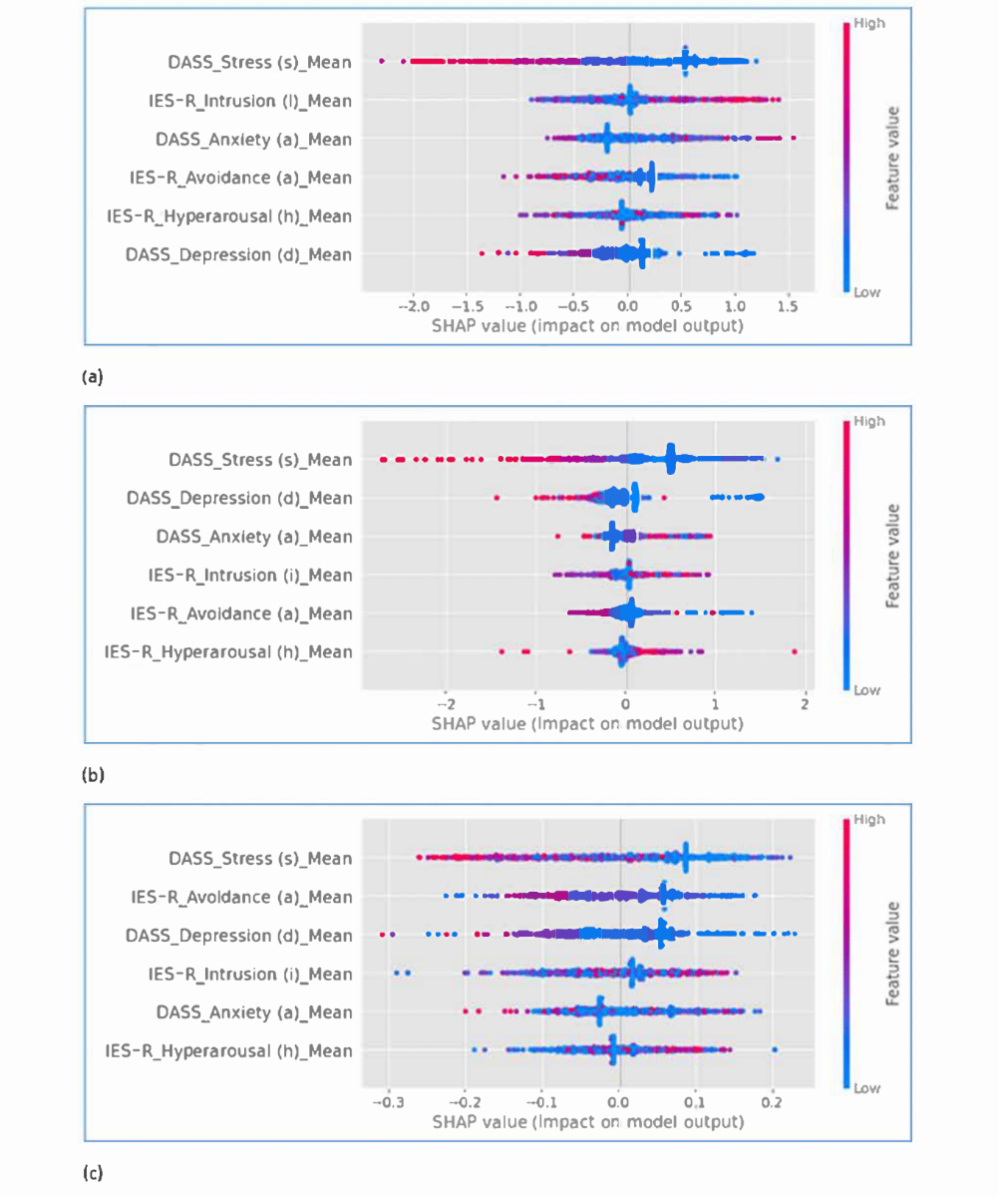
SHAP value plots: (a) LightGBM model, (b) GradientBoost model, and (c) RandomForest model. LightGBM: Light Gradient Boosting Machine; SHAP: Shapley Additive Explanation.

Figures 7a-7c represent the SHAP value plots. There is a corresponding SHAP value of each independent variable computed for all the data points, providing the local interpretation for understanding individual predictions. The magnitude of a SHAP value represents the impact of each independent variable in deviating the predicted values from the base value [24]. The base value is the average of the model outputs for the entire training data set. The sign of a SHAP value represents the directional force that increases (positive sign) or decreases (negative sign) the prediction away from the base value.

SHAP values can be plotted to provide a global interpretation of the model in understanding the general model behavior based on the model features. The red and blue dots, as shown in SHAP value plots (Figures 7a-7c), represent the higher and lower magnitude levels (referred to as feature values), respectively, as compared with the predicted values contributed by all individual data points. The amount of influence in both positive and negative directions of a variable is shown by the spread of the dots from the center. The variable with the widest spread of red dots (or appearing to have more red dots) than other variables is the most influential variable. As shown in the plots, variables are positioned from top to bottom sorted in the order of their importance.

Take Figure 7a for the LightGBM model as an example. Most of the red dots for DASS-21 stress scores are on the left side, which is the region for negative SHAP values, and are most spread out compared to other variables. This indicates that the DASS-21 stress item is the first variable on the list having the highest amount of influence over the LightGBM model, and stress has a negative relationship (the lower the stress, the higher the impact on the model prediction). Stress is placed right at the top in the plot corresponding with its rank of importance. This phenomenon about the stress of nurses was consistent in the SHAP value plots of all the 3 models. As shown in Table 6, stress in the negative direction was ranked first for all the models, and we could affirm that the most distinctive psychological characteristic of nurses was the lower stress level compared to doctors and nonmedical health care workers.

**Table 6:** Global interpretation of SHAP^a^ value plots.

Rank^b^ of variable influence	LightGBM^c^ model	GradientBoost model	RandomForest model
1	Stress (–)^d^	Stress (–)	Stress (–)
2	Intrusion (+)^d^	Depression (–)	Avoidance (–)
3	Anxiety (+)	Anxiety (+)	Depression (–)
4	Avoidance (–)	Intrusion (+)	Intrusion (+)
5	Hyperarousal (+)	Avoidance (–)	Anxiety (+)
6	Depression (–)	Hyperarousal (+)	Hyperarousal (+)

^a^SHAP: Shapley Additive Explanation.

^b^Rank 1 is most influential. Variables of each model were ranked and filled in accordingly in the table. The variable with the widest spread of red dots was ranked 1.

^c^LightGBM: Light Gradient Boosting Machine.

^d^The “–” and “+” represent the direction of force in value prediction and correspond with the right or left region, respectively, where most red dots fall within the SHAP value plots. The “–” and “+” signs of a variable are the same for all the models.

## Discussion

### Principal Findings

The principal findings of our study are twofold. First, during the early phase of COVID-19 in the Asia-Pacific region, nurses were least psychologically affected than other health care workers. This was evidenced by their relatively higher percentages of normal or unchanged psychological distress symptoms relative to other health care workers. Additionally, among health care workers with no psychological distress symptoms, nurses constituted a higher proportion than doctors and nonmedical health care workers. Notably, despite the most demanding job nature (ie, greater exposure and longer time with patients), nurses showed the lowest level of stress, which is the most distinctive psychological outcome characteristic compared to other health care workers. Second, nurses in Vietnam showed the highest level of depression, stress, intrusion, avoidance, and hyperarousal symptoms compared to those in Singapore, Malaysia, and Indonesia. Nurses in Singapore had the highest level of anxiety. The differing patterns of psychological outcomes among nurses are probably related to different contexts, cultures, and points in the pandemic curve.

Our finding that amongst health care workers, frontline nurses were least psychologically affected is consistent with a previous study [25]. Li et al [25] reported that frontline nurses had lower vicarious traumatization scores relative to nonfrontline nurses and the general public. Furthermore, our finding is also consistent with a previous study conducted during the SARS pandemic [26], which reported that doctors were 1.6 times more likely to experience psychiatric symptoms than nurses. The authors attributed higher rates of doctors’ anxiety to the need for maintaining a prolonged hypervigilant state in diagnosing SARS cases. By comparison, a rapid review conducted during the early phase of COVID-19 suggested that nurses may be at a higher risk for adverse mental health outcomes than doctors [27]. However, the authors acknowledged that confounding factors in the studies were not robustly addressed. The reason why nurses were least psychologically affected could be explained by their job scopes. The nature of the nursing profession in clinical settings requires teamwork. An integrative review suggested that positive teamwork has a significant correlation with mental resilience [28]. It is possible that despite the demanding workload during the pandemic, nurses have good mental resilience as they work in teams.

Although we believe that the differing patterns of psychological outcomes amongst nurses in various Asia-Pacific countries could be related to the magnitude of the pandemic in those countries, despite having the lowest volume of cases during the study period, nurses in Vietnam showed the highest level of depression, stress, intrusion, avoidance, and hyperarousal relative to nurses in Singapore, Malaysia, and Indonesia. This is in line with a previous study in Vietnam that reported a higher level of psychological distress in nurses than doctors, attributing the findings to their job nature and demands [29]. Singapore had the highest case volume, which might have contributed to the relatively higher anxiety level of nurses.

Although nurses in India showed the highest level of hyperarousal, the data for India were not accounted for in our conclusions due to the differing psychological response pattern exhibited amongst the nurses in India. The highest level of hyperarousal in nurses in India might be due to the larger proportion of younger nurses (two-third being <35 years old). A younger age is considered a risk factor for PTSD symptoms [30]. In view of fewer older and experienced nurses in India, younger nurses are likely to experience higher levels of hyperarousal due to the lack of supervision support, guidance, and leadership. It is also reported that a large number of female nurses across India are emigrants from states in South India, especially Kerala [31]. The emigrated nurses usually live alone in their cities of employment. With the lack of support from family members, they could not have psychologically managed themselves well in facing the work challenges posed by the COVID-19 pandemic.

During the early phase of the pandemic curve, relative to other health care workers, although nurses seemed to cope better psychologically, yet approximately three-fifth of the nurses reported some psychological distress. Notably, this proportion was lower than doctors (approximately three-fourth) and nonmedical health care workers (two-third). This highlights significant psychological outcomes of COVID-19, for which effective coping strategies are essential to bolster psychological resilience in all health care workers, especially doctors and nonmedical health care workers.

During the early COVID-19 phase, health care workers had coped with the pandemic by holding on to their values. This is consistent with the meaning-making coping style postulated by Park and Folkman [32]. That is, health care workers coped by making meaning of their work with altruistic beliefs and goals in the battle against the pandemic. This led to psychological adaption and personal growth to occur early and possibly within 2 weeks [33]. Nurses reported their personal growth under the crisis (eg, gratitude, a stronger sense of professional identity, and self-reflection). Such growth promoted positive emotions and psychological adaptation. Moreover, health care workers reported improved mood over the 2 weeks’ duration, due to acquiring more knowledge about COVID-19 [33]. This seems to concur with the decline in the frequency of mental health problems of Chinese health care workers over time [34,35].

With almost 2 years into the pandemic, the International Council of Nurses found that 20% of National Nurses Associations reported increased rates of nurses leaving the profession in 2020, likely due to the pandemic [36]. Accordingly, a recent scoping review reported that nurses will be under an increased risk for stress, burnout, and depression during the pandemic, where younger female nurses, with less clinical experience, are more vulnerable to adverse mental health outcomes [37]. Catton and Iro [38], nurses in WHO and the International Council of Nurses (ICN), recently called for an investment in the augmentation of the nursing profession since the availability of adequate nursing staff can reduce inpatient admissions and hospital stays [39], a dire situation that most countries are currently facing.

In view of the aforementioned worrying trend, and the fact that health care workers prefer self-help rather than seeking professional help [40,41], we recommend self-care coping strategies, such as self-reflection, reinforcing internal values, aerobic exercises, and support from religious organizations, peers, and family. These strategies were helpful during the previous SARS and Ebola epidemics [42-45]. Similarly, digital interventions, such as computerized resilience training over a medium-long course duration (12-17 sessions), may be useful to build up health care workers’ resilience [46]. Furthermore, peer-led cognitive behavioral therapy group programs may also assist health care workers to cope psychologically [47]. Moreover, a structured peer support program through caring mentorship and self-reflection practice would be practical and useful for health care workers to continue to strive in coping with the pandemic [48,49].

### Strengths

The novelty of our study is twofold. First, we used machine learning techniques and visualizations to discover trends. This novel machine learning approach predicts whether a health care worker is a nurse from a set of psychological scores. Although versatile models (eg, decision tree, neural network) are desirable for accuracy, many hesitate to adopt them, as the model interpretation of the independent variables is challenging. In our study, decision tree–based models were adopted based on the fact that SHAP could explain the models and pick out the most distinctive characteristic. The use of SHAP in unravelling distinctive characteristics manifests the power of the SHAP algorithm [50]. With SHAP, the advantages of decision tree–based machine learning models in requiring less data processing effort and a not too large data set can be widely applied in situations in which the underlying principles of the relationship between variables are unknown and a quick solution is required. These are the limitations that a traditional statistical approach cannot overcome. For example, the statistical *t* test will not lead to a correct conclusion if the assumptions of the *t* test are not present (eg, 1 of the assumptions is that data points should follow a normal distribution). The *P* value of the 2-sample *t* test for the DASS-21 stress item was the lowest among all psychological distress characteristics (ie, the highest level of difference) for the comparisons between nurses and doctors and between nurses and nonmedical health care workers (Table 4). This is consistent with our SHAP result, which shows a significant lower level of stress amongst nurses (Figures 7a-7c). The same result cannot be arrived at if the assumptions of the *t* test are not met. Like the *t* test (one-tailed), SHAP is capable of indicating the direction of influence (positive or negative) for each psychological distress characteristic. In addition, SHAP can present the level of contrast in graphical form. The notable advantage of SHAP is the model interpretation at both global and local levels, thus constituting a full suite of explanations for the gap between the overall average value and the model prediction. Such user-friendly interpretation style allows wide application of the SHAP technique in health care research. Second, to better understand the psychological outcomes of health care workers from a wider range of the Asia-Pacific region, we recruited a large sample from several countries. These health care workers have different occupational roles and were functioning in both medical and nonmedical roles during the early phase, which was the most stressful period of the pandemic. Understanding their psychological outcomes will help the health care industry to better prepare for future pandemics. In urgent situations like the current pandemic, where the presentations evolved rapidly, without any precedent case, machine learning can be a faster and efficient option to analyze data for identifying patterns and taking appropriate actions in a timely manner.

### Limitations

Some limitations of our study merit acknowledgement. First, there are no similar mental health data prior to COVID-19 for comparison. This limited us to provide insight into the direct impact of COVID-19 on health care workers’ psychological outcomes. Second, we used a cross-sectional survey with participant self-report questionnaires; hence, it was difficult to accurately assess mental health problems, due to a lack of specialist verification. Third, we did not have a follow-up study design, which could evaluate long-term mental health outcomes and coping. Future studies should include a better study design using clinical verification to further assess cases with significant psychological distress and follow them up to investigate the long-term psychological outcomes.

### Conclusion

In conclusion, during the early phase of COVID-19, nurses were least psychologically affected as they have better teamwork compared to other health care workers. Different contexts, cultures, and points in the pandemic curve may have also contributed to differing patterns of psychological outcomes amongst nurses in various Asia-Pacific countries. With no real end in sight for the current pandemic, along with their multidisciplinary colleagues, nurses worldwide are persevering to fight this war. Nurses require targeted psychological support dependent on regions, contexts, cultures, and points in the pandemic curve. Similarly, active outreach and targeted interventions should be provided to nonmedical health care workers and medical doctors to support their psychological well-being. To win this battle, it is crucial for all health care workers to hold on to their values, practice self-care, and render peer support to bolster psychological resilience for effective coping.

## Appendix

Multimedia Appendix 1 Supplementary materials: Tables S1 and S2.

## Abbreviations

AUROC: area under the receiver operating characteristic curve
DASS-21: Depression Anxiety Stress Scale
IES-R: Impact of Events Scale-Revised
LightGBM: Light Gradient Boosting Machine
PTSD: posttraumatic stress disorder
SHAP: Shapley Additive Explanation
WHO: World Health Organization
