# Supplementary material for: Investigating Psychological Differences Between Nurses and Other Health Care Workers From the Asia-Pacific Region During the Early Phase of COVID-19: Machine Learning Approach
Source: JMIR Nurs. 2022 Jun 1;5(1):e32647. doi: 10.2196/32647 (PMC9162133; doi:10.2196/32647)
Supplement: Multimedia Appendix 1 [file nursing_v5i1e32647_app1.docx]

**Supplementary Paper for**

- **Investigating Psychological Differences between Nurses and Other Healthcare Workers from Asia-Pacific Region during the Early Phase of the Coronavirus Disease 2019 (COVID-19): A Machine Learning Approach**

YanHong Dong^1,2^, PhD, Mei Chun Yeo^3^, M. Eng, M. Tech, Xiang Cong Tham^1^, MPH, Rivan Danuaji^4^, MD, Thang H Nguyen^5^, MD, Arvind K Sharma^6^, MD, Komalkumar RN^7^, MD, Meenakshi RV^8^, MD, Tai Mei-Ling Sharon^9^, MD, Aftab Ahmad^10^, MRCP, Benjamin YQ Tan^11^, MBBS, Roger C Ho^12^, MRCP, Matthew Chin Heng Chua^3^, PhD, Vijay K. Sharma^2,11^, MRCP

^1^ Alice Lee Centre for Nursing Studies, Yong Loo Lin School of Medicine, National University of Singapore, Singapore

^2^ Department of Medicine, Yong Loo Lin School of Medicine, National University of Singapore, Singapore

^3^ Institute of Systems Science, National University of Singapore, Singapore

^4^ Dr Moewardi Hospital Surakarta, Jawa Tengah, Indonesia

^5^ Cerebrovascular Disease Department, 115 People’s Hospital, Ho Chi Minh City, Vietnam

^6^ Zydus hospital Ahmedabad, India

^7^ Yashoda Hospital, Secunderabad, India

^8^ Senthil Multi Specialty Hospital, Erode, India

^9^ University of Malaya, Kuala Lumpur, Malaysia

^10^ Department of Neurology, Ng Teng Fong General Hospital, Singapore

^11^ Division of Neurology, Department of Medicine, National University Health System, Singapore

^12^ Department of Psychological Medicine, National University Hospital, Singapore and Yong Loo Lin School of Medicine, National University of Singapore, Singapore

***Corresponding Author:**

Dr. YanHong Dong, Alice Lee Centre for Nursing Studies, Yong Loo Lin School of Medicine, National University of Singapore, Clinical Research Centre, Block MD11, Level 2, 10 Medical Drive, Singapore 117597.

Phone: +65 65168686; Email: [nurdy@nus.edu.sg](mailto:nurdy@nus.edu.sg)

**Supplementary Paper**

**Results**

**Differences between Nurses, Doctors and Non-Medical Healthcare Workers**

The sets of statistics including the data for India are provided in this supplementary paper. Tables 7 and 8 in this supplementary paper corresponds with Tables 4 and 5 of the main paper respectively.

**Table S1.** Mean Scores of Psychological Distress Characteristics by Profession and t-test results (All five countries, including India)

| **Type** | **Depression** | **Anxiety** | **Stress** | **Intrusion^** | **Avoidance^** | **Hyperarousal^** |
| --- | --- | --- | --- | --- | --- | --- |
| Scores by Profession | | | | | | |
| **Overall *** | 0.2340 | 0.2391 | 0.3385 | 0.3098 | 0.3100 | 0.2811 |
| **Nurses** | 0.1736 | 0.1788 | 0.2515 | 0.2783 | 0.2834 | **0.2997** |
| **Doctors** | **0.2994** | **0.2650** | **0.4025** | 0.3013 | 0.2908 | 0.2653 |
| **Non-medical** | **0.2524** | **0.2873** | **0.3879** | **0.3519** | **0.3545** | 0.2722 |
| Two-tail p-values of two-sample t-test for comparing mean values, alpha 0.05 | | | | | | |
| **Nurses vs**  **Doctors** | 1.27E-05  (diff) | 4.83E-04  (diff) | 2.82E-06  (diff) | 0.3719  (no diff) | 0.7902  (no diff) | 0.1803  (no diff) |
| **Nurses vs**  **Non-medical** | 0.0011  (diff) | 6.68E-07  (diff) | 1.60E-07  (diff) | 0.0032  (diff) | 0.0095  (diff) | 0.2383  (no diff) |
| - “Score” refers to the average of mean scores or normalised mean scores. - If a p-value of two-sample t-test is lesser than 0.05, it represents that there is a difference in the mean scores or normalised mean scores (denoted by “diff”). Otherwise, there is no difference (denoted by “no diff”). - Normalised values of multiplying by 3 and dividing by 4 are adopted for IES-R subscales (namely Intrusion, Avoidance, and Hyperarousal, which are marked with ^). This is to make IES-R scores (from 0 to 4) to be in the same scale as DASS-21 (from 0 to 3). - Numbers in bold and in dark background shade are the mean score by profession that are higher than the overall respective mean scores (Row marked with *). | | | | | | |

**Table S2.** Psychological Distress Severity (All five countries, including India)

| **Severity**  **Category** | **Depression**  **n (%)** | **Anxiety**  **n (%)** | **Stress**  **n (%)** | **Intrusion**  **n (%)** | **Avoidance**  **n (%)** | **Hyperarousal**  **n (%)** |
| --- | --- | --- | --- | --- | --- | --- |
| **All (including Nurses, Doctors, and Non-medical Healthcare Workers)** | | | | | | |
| **Normal /**  **Not at all** | 1001  (89.2) | 953  (84.9) | 1054  (93.9) | 997  (88.9) | 972  (86.6) | 998  (88.9) |
| **Mild / A little bit, and above** | 121  (10.8) | 169  (15.1) | 68  (6.1) | 125  (11.1) | 150  (13.4) | 124  (11.1) |
| **Nurses** | | | | | | |
| **Normal /**  **Not at all** | 406  (92.7) | 399  (91.1) | 424  (96.8) | 395  (90.2) | 389  (88.8) | 378  (86.3) |
| **Mild / A little bit, and above** | 32  (7.3) | 39  (8.9) | 14  (3.2) | 43  (9.8) | 49  (11.2) | 60  (13.7) |
| **Doctors** | | | | | | |
| **Normal /**  **Not at all** | 249  (84.4) | 238  (80.7) | 271  (91.9) | 264  (89.5) | 259  (87.8) | 265  (89.8) |
| **Mild / A little bit, and above** | 46  (15.6) | 57  (19.3) | 24  (8.1) | 31  (10.5) | 36  (12.2) | 30  (10.2) |
| **Non-medical Healthcare Workers** | | | | | | |
| **Normal /**  **Not at all** | 346  (88.9) | 316  (81.2) | 359  (92.3) | 338  (86.9) | 324  (83.3) | 355  (91.3) |
| **Mild / A little bit, and above** | 43  (11.1) | 73  (18.8) | 30  (7.7) | 51  (13.1) | 65  (16.7) | 34  (8.7) |
| DASS-21 severity categories are based on 2 times of sum of subscale scores   - Depression 🡪 mild and above: >= 10 - Anxiety 🡪 mild and above: >= 8 - Stress 🡪 mild and above: >= 15   IES-R severity categories are based on mean of subscale scores   - Intrusion, Avoidance, Hyperarousal 🡪 a little bit and above: >= 1 | | | | | | |
